# Neuropeptide Y1 Receptor Regulates Glucocorticoid-Induced Inhibition of Osteoblast Differentiation in Murine MC3T3-E1 Cells via ERK Signaling

**DOI:** 10.3390/ijms17122150

**Published:** 2016-12-21

**Authors:** Wei Yu, Chao Zhu, Wenning Xu, Leisheng Jiang, Shengdan Jiang

**Affiliations:** Department of Orthopedic Surgery, Xinhua Hospital, Shanghai Jiaotong University School of Medicine, Shanghai 200092, China; halouyuwei@sjtu.edu.cn (W.Y.); chaozhu@sjtu.edu.cn (C.Z.); winningxu@sjtu.edu.cn (W.X.)

**Keywords:** glucocorticoid, NPY (neuropeptide Y), neuropeptide Y1 receptor, osteoblast

## Abstract

High dose glucocorticoid (GC) administration impairs the viability and function of osteoblasts, thus causing osteoporosis and osteonecrosis. Neuropeptide Y1 receptor (Y1 receptor) is expressed in bone tissues and cells, and regulates bone remodeling. However, the role of Y1 receptor in glucocorticoid-induced inhibition of osteoblast differentiation remains unknown. In the present study, osteoblastic cell line MC3T3-E1 cultured in osteogenic differentiation medium was treated with or without of 10^−7^ M dexamethasone (Dex), Y1 receptor shRNA interference, Y1 receptor agonist [Leu^31^, Pro^34^]-NPY, and antagonist BIBP3226. Cell proliferation and apoptosis were assessed by cell counting kit-8 (CCK-8) assay and cleaved caspase expression, respectively. Osteoblast differentiation was evaluated by Alizarin Red S staining and osteogenic marker gene expressions. Protein expression was detected by Western blot analysis. Dex upregulated the expression of Y1 receptor in MC3T3-E1 cells associated with reduced osteogenic gene expressions and mineralization. Blockade of Y1 receptor by shRNA transfection and BIBP3226 significantly attenuated the inhibitory effects of Dex on osteoblastic activity. Y1 receptor signaling modulated the activation of extracellular signal-regulated kinases (ERK) as well as the expressions of osteogenic genes. Y1 receptor agonist inhibited ERK phosphorylation and osteoblast differentiation, while Y1 receptor blockade exhibited the opposite effects. Activation of ERK signaling by constitutive active mutant of *MEK1* (caMEK) abolished Y1 receptor-mediated Dex inhibition of osteoblast differentiation in MC3T3-E1 cells. Taken together, Y1 receptor regulates Dex-induced inhibition of osteoblast differentiation in murine MC3T3-E1 cells via ERK signaling. This study provides a novel role of Y1 receptor in the process of GC-induced suppression in osteoblast survival and differentiation.

## 1. Introduction

Glucocorticoids (GCs) are extensively used as immunosuppressive and anti-inflammatory drugs for various disorders including autoimmune diseases and inflammatory [1,2]. Excessive or long-term administration of glucocorticoids causes several adverse effects on the bones, including osteoporosis and osteonecrosis [3,4,5]. Glucocorticoids inhibit the survival and differentiation capacity of osteoblasts, which is considered a prominent mechanism in the process of GC-induced bone loss [6]. Previous studies have shown that induction of cell apoptosis or autophagy contributes to glucocorticoid-induced loss of bone cell viability [4,7]. Glucocorticoids disturb the process of osteogenic differentiation by shifting bone marrow-derived stem cells (BMSCs) from osteoblast lineage towards adipocyte lineage in bone microenvironments [8]. However, the precise mechanisms by which glucocorticoids regulate the proliferation and differentiation pathways in osteoblasts are still unknown.

Neuropeptide Y (NPY), a 36-amino-acid peptide abundantly expressed in the central nervous systems, is found to play an important role in the regulation of bone metabolism as well as the modulation of food intake and energy balance [9]. Among the five known receptors (Y1, Y2, Y4, Y5, and Y6 receptors) for NPY, peripheral Y1 and central Y2 receptors have been revealed to regulate bone remodeling [9,10,11]. In vivo, germ-line deletion of Y1 or Y2 receptor increases the bone mass of mice owing to increased osteoblasts activity and bone formation [12,13,14]. Blockade of Y1 receptor by its antagonist had similar effects on bone remodeling [15]. In vitro, NPY treatment decreased the proliferation and differentiation of osteoblasts via activation of the Y1 receptor [11,16]. Y1 receptor knockdown enhanced osteogenic differentiation in bone-marrow mesenchymal stem cells [11]. In addition, the expression of Y1 receptor, but not Y2 receptor, has been detected in the osteoblastic cells lining the bone surface and in calvaria-derived osteoblasts [9,12]. Osteoblast-specific Y1 receptor deletion led to increased bone mass in mice, similar to the results of Y1 receptor germ-line deletion, confirming the peripheral effects of Y1 receptor on bone formation through direct action on osteoblasts [17]. These results demonstrated the expression of Y1 receptor in osteoblasts, and indicated that the Y1 receptor might play a negative role in bone metabolism. 

Crosstalk between NPY system and glucocorticoid is found in the regulation of various functions in different cells [18,19]. Moreover, a recent study demonstrated that increased NPY expression was associated with glucocorticoid-induced bone loss and marrow adiposity in mice, whereas NPY deletion protected bone tissue against glucocorticoid-induced deterioration [20]. The Y1 receptor is the main receptor for NPY; however, its role in the glucocorticoid-induced suppression of osteoblast differentiation at the cell level has not yet been defined. This study explored the role of the Y1 receptor in dexamethasone-induced suppression of osteoblast differentiation, and further investigated whether regulation of Y1 receptor function influenced the differentiation of osteoblastic cells with dexamethasone treatment. The cellular signaling involved in this process was also explored. 

## 2. Results

### 2.1. Upregulation of Y1 Receptor Expression by Dexamethasone

**Figure 1:**
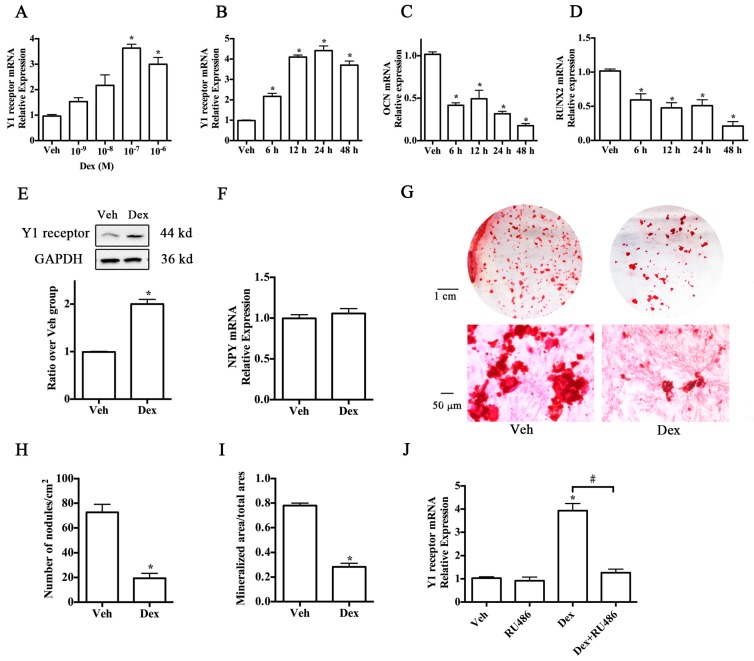
Dex upregulated the expression Y1 receptor in association with suppression of osteoblast differentiation in MC3T3-E1 cells; (**A**) Dex enhanced the expression of Y1 receptor in a dose-dependent manner; (**B**–**D**) 10^−7^ M Dex increased the mRNA expression of Y1 receptor but decreased that of osteocalcin (OCN) and runt-related transcription factor 2 (RUNX2) in a time-dependent manner; (**E**) 10^−7^ M Dex increased the level of Y1 receptor protein expression; (**F**) 10^−7^ M Dex did not affect the level of NPY mRNA in osteoblasts; (**G**) Alizarin Red S staining; (**H**,**I**) osteoblast mineralization analysis; (**J**) 10^−5^ M RU486 (glucocorticoid receptor antagonist) abolished the promoting effects of Dex on Y1 receptor mRNA expression; MC3T3-E1 cells in osteogenic differentiation media were treated with or without 10^−7^ M dexamethasone for one day and 21 days; The expression profiles of mRNA and protein were detected by real-time PCR and Western blot, respectively; Mineralization of MC3T3-E1 cells was determined by Alizarin Red S staining on day 21; Data are presented as means ± SEM; * *p* < 0.05 (compared to vehicle); # *p* < 0.05 (compared to Dex); Veh: vehicle; Dex: dexamethasone; GAPDH: glyceraldehyde 3-phosphate dehydrogenase.

To examine the role of the Y1 receptor in the glucocorticoid-induced suppression of osteoblast differentiation, we first detected the expression of Y1 receptor in MC3T3-E1 cells with or without dexamethasone (Dex) treatment in osteogenic differentiation media. The results of real-time PCR demonstrated that the expression of Y1 receptor was upregulated by dexamethasone in a dose-dependent manner (Figure 1A), with 10^−7^ M being the most effective concentration. Application of 10^−7^ M dexamethasone to MC3T3-E1 cells for 48 h caused a significant increase of Y1 receptor mRNA expression (Figure 1B) in parallel with a decreased level of osteocalcin (OCN) and runt-related transcription factor 2 (RUNX2) expression (Figure 1C,D). Similarly, Western blot demonstrated that cells with 10^−7^ M dexamethasone treatments for 24 h displayed a high level of Y1 receptor expression (Figure 1E). To investigate whether Dex activated Y1 receptor through an NPY-independent pathway, we monitored the NPY mRNA levels after Dex treatment. As shown in Figure 1F, 10^−7^ M Dex did not affect NPY mRNA expression in cell cultures. Alizarin Red S staining at day 21 showed that cells with dexamethasone treatment exhibited weaker mineralization ability than the controls (Figure 1G). Fewer bone nodules and smaller mineralized matrix areas were found in Dex groups (Figure 1H,I) in comparison with the control groups. In addition, to investigate whether the glucocorticoid receptor was involved in glucocorticoid-induced upregulation of Y1 receptor expression, we added glucocorticoid receptor antagonist RU486 (10^−5^ M) to the culture media and found that RU486 significantly reduced glucocorticoid-induced Y1 receptor expression in MC3T3-E1 cells (Figure 1J). 

### 2.2. Knockdown of the Y1 Receptor Enhanced Osteoblast Differentiation

**Figure 2:**
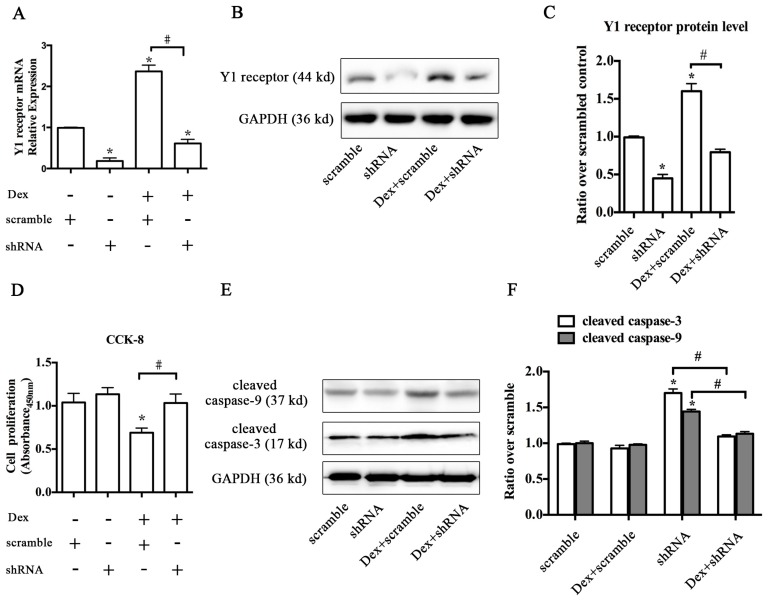
Knockdown of the Y1 receptor attenuated Dex-induced inhibition of cell proliferation and alleviated Dex-induced apoptosis in osteoblastic MC3T3-E1 cells; (**A**) Silencing of the Y1 receptor by shRNA plasmid decreased the baseline and Dex-induced Y1 receptor expression at the mRNA and (**B**,**C**) protein levels. (**D**) Silencing of the Y1 receptor attenuated the effects of Dex on cell proliferation and (**E**) cell apoptosis in osteoblastic MC3T3-E1 cells; (**E**,**F**) MC3T3-E1 cells treated with Dex exhibited high levels of cleaved caspase 3 and cleaved caspase 9, which were decreased following Y1 receptor interference; MC3T3-E1 cells were transfected with a scrambled control or shRNA plasmid, treated with or without 10^−7^ M Dex in osteogenic differentiation media for one day; Cell proliferation was determined using CCK-8 assay and cell apoptosis was detected by immunoblotting of cleaved caspase 3 and cleaved caspase 9. Data are presented as means ± SEM; * *p* < 0.05 (compared to vehicle); # *p* < 0.05 (compared to Dex); Veh: vehicle; Dex: dexamethasone; GAPDH: glyceraldehyde 3-phosphate dehydrogenase; CCK-8: cell counting kit-8.

To test whether Y1 receptor inhibition influenced Dex-induced suppression of osteoblast differentiation in MC3T3-E1 cells, we silenced the Y1 receptor using shRNA interference. The level of Y1 receptor mRNA was significantly decreased after treatment with shRNA plasmid targeting Y1 receptor, suggesting a high efficiency of shRNA interference (Figure 2A). The results of Western blot also showed that shRNA interference decreased the previous abundance of Y1 receptor in MC3T3-E1 cells, while the level of glyceraldehyde 3-phosphate dehydrogenase (GAPDH) was not altered (Figure 2B,C). Knockdown of Y1 receptor attenuated the inhibitory effects of Dex on the proliferation ability of MC3T3-E1 cells (Figure 2D). Activation of caspases has been shown to contribute to apoptosis in various types of cells [21,22]. Thus, we evaluated cell apoptosis by detecting the levels of cleaved caspase 3 and cleaved caspase 9, two key molecules involved in apoptosis process. Dex significantly increased the levels of cleaved caspase 3 and cleaved caspase 9, whereas Y1 receptor knockdown reversed this trend (Figure 2E,F). 

**Figure 3:**
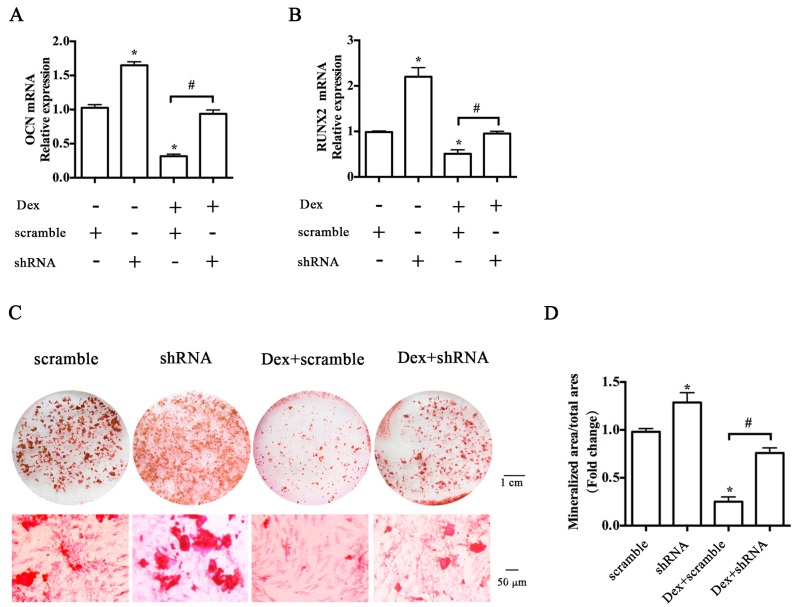
Knockdown of Y1 receptor attenuated the Dex-induced suppression of osteoblast differentiation in MC3T3-E1 cells; (**A**) Knockdown of Y1 receptor restored the decreased levels of runt-related transcription factor 2 (RUNX2) and (**B**) osteocalcin (OCN) expression after Dex treatment; (**C**) Knockdown of Y1 receptor attenuated the inhibitory effects of Dex on mineralized matrix formation and (**D**) on mineralized areas in MC3T3-E1 cells; Representative results of Alizarin Red S staining in MC3T3-E1 cells on day 21 are shown; MC3T3-E1 cells were transfected with a scrambled control or shRNA plasmid, treated with or without 10^−7^ M Dex in osteogenic differentiation media for 21 days; Mineralization of MC3T3-E1 cells was determined by Alizarin Red S staining; Data are presented as means ± SEM; * *p* < 0.05 (compared to vehicle); # *p* < 0.05 (compared to Dex); Veh: vehicle; Dex: dexamethasone.

Furthermore, the inhibitory effects of Dex on the expression of RUNX2 and osteocalcin (OCN), two osteogenic marker genes, were reversed by Y1 receptor shRNA (Figure 3A,B). Alizarin Red S staining at 21 days demonstrated that cells with Y1 receptor shRNA interference significantly attenuated the Dex-induced reduction of mineralized matrix areas in MC3T3-E1 cells (Figure 3C,D). Notably, Y1 receptor shRNA alone also enhanced the baseline of osteogenic marker genes expressions and mineralization of cell cultures. Taken together, knockdown of Y1 receptor by shRNA interference enhanced osteoblast differentiation, and restored cell survival and differentiation in osteoblastic MC3T3-E1 cells following Dex treatment. 

### 2.3. Y1 Receptor Antagonist Regulated the Mineralization of MC3T3-E1 Cells

**Figure 4:**
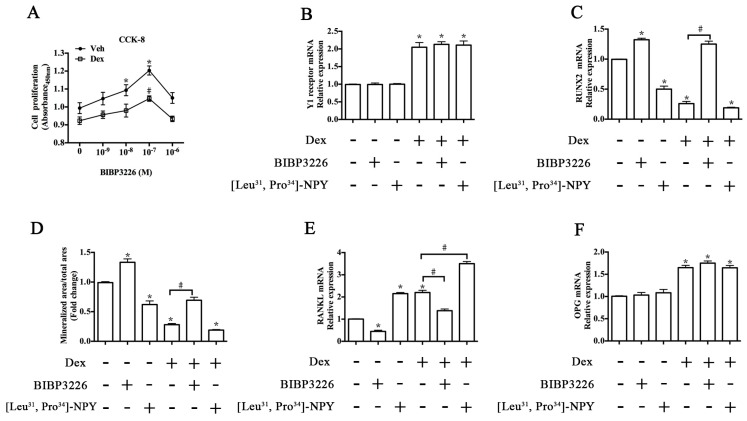
BIBP3226 and [Leu^31^, Pro^34^]-NPY regulated the proliferation and mineralized capacity of MC3T3-E1 cells; (**A**) BIBP3226 alleviated the inhibitory effects of Dex on cell proliferation in a dose-dependent manner; (**B**) Neither BIBP3226 (10^−7^ M) nor [Leu^31^, Pro^34^]-NPY (10^−7^ M) affected the expression of Y1 receptor mRNA in MC3T3-E1 cells; (**C**) BIBP3226 (10^−7^ M) attenuated the adverse effects of Dex on runt-related transcription factor 2 (RUNX2) expression and (**D**) on mineralization capacity in MC3T3-E1 cells; (**C**) [Leu^31^, Pro^34^]-NPY (10^−7^ M) reduced the baseline of RUNX2 expression and of (**D**) mineralization in MC3T3-E1 cells, strengthening the adverse effects of Dex on osteoblast differentiation; (**E**) BIBP3226 decreased while [Leu^31^, Pro^34^]-NPY increased RANKL expression in MC3T3-E1 cells; (**F**) Neither BIBP3226 nor [Leu^31^, Pro^34^]-NPY affected the expression of OPG mRNA; MC3T3-E1 cells treated with or without BIBP3226, [Leu^31^, Pro^34^]-NPY, and Dex were cultured in osteogenic differentiation media for one day or 21 days; Mineralization of MC3T3-E1 cells was determined by Alizarin Red S staining; * *p* < 0.05 (compared to vehicle); # *p* < 0.05 (compared to Dex); Veh: vehicle; Dex: dexamethasone; RANKL: receptor activator of nuclear factor kappa-B ligand; OPG: osteoprotegerin.

We then investigated the effects of Y1 receptor signaling regulation on the osteoblast differentiation in MC3T3-E1 cells by using the pharmacological Y1 receptor agonist [Leu^31^, Pro^34^]-NPY and antagonist BIBP3226. BIBP3226 at a concentration of 10^−6^–10^−9^ M significantly attenuated the Dex-induced inhibition of cell proliferation at 24 h (Figure 4A). 10^−7^ M was the optimal concentration for BIBP3226 to promote the cell proliferation, thus this concentration of BIBP3226 was adopted in the subsequent experiments. Neither [Leu^31^, Pro^34^]-NPY nor BIBP3226 affects the baseline and Dex-mediated transcription of Y1 receptor (Figure 4B). Compared to the Dex group, BIBP3226 treatment significantly alleviated the inhibitory effects of Dex on RUNX2 expression (Figure 4C) and mineralization (Figure 4D). BIBP3226 also reduced the level of the receptor activator of nuclear factor kappa-B ligand (RANKL) expression induced by Dex (Figure 4E). In contrast, [Leu^31^, Pro^34^]-NPY suppressed osteoblast differentiation and enhanced the biological effects of Dex on RUNX2, RANKL expression, and mineralization of MC3T3-E1 cells. Notably, neither [Leu^31^, Pro^34^]-NPY nor BIBP3226 treatment affected the induction of osteoprotegerin (OPG) expression by glucocorticoid (Figure 4F). BIBP3226 treatment attenuated the glucocorticoid-induced apoptosis of MC3T3-E1 cells (data not shown). 

### 2.4. ERK Signaling Participated in Y1 Receptor-Mediated Suppression of Osteogenic Differentiation 

**Figure 5:**
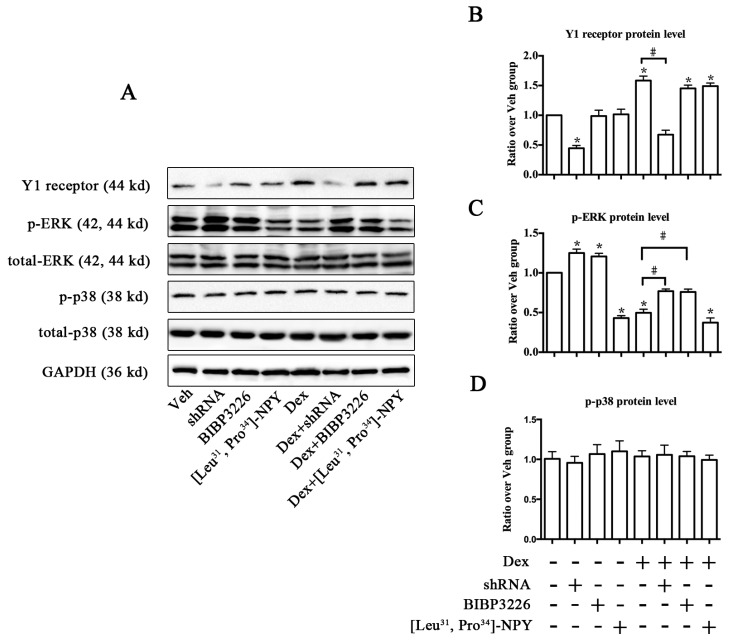
Y1 receptor regulated the phosphorylation of ERK signaling pathway in MC3T3-E1 cells; (**A**) Representative results of Western blot were shown; (**B**) Neither BIBP3226 nor [Leu^31^, Pro^34^]-NPY affected the expression profiles of Y1 receptor protein; (**C**) Blockade of Y1 receptor by RNA interference and antagonist BIBP3226 increased the baseline and Dex-modulated level of ERK phosphorylation, whereas [Leu^31^, Pro^34^]-NPY decreased ERK phosphorylation in MC3T3-E1 cells; (**D**) The expression of p38 was not affected by Y1 receptor modulation; * *p* < 0.05 (compared to vehicle); # *p* < 0.05 (compared to Dex); EKR: extracellular signal-regulated kinases; GAPDH: glyceraldehyde 3-phosphate dehydrogenase; Veh: vehicle; Dex: dexamethasone.

We further explored the signaling pathways involved in Y1 receptor regulation of osteoblast differentiation. Western blot (Figure 5A) demonstrated that Y1 receptor knockdown by shRNA significantly decreased the expression profiles of Y1 receptor protein, whereas [Leu^31^, Pro^34^]-NPY and BIBP3226 treatments did not affect Dex-induced Y1 receptor expression (Figure 5B). On the basis that Y1 receptor signaling regulated bone metabolism, and because ERK (extracellular signal-regulated kinases) and/or p38 signaling mediated various biological reactions in osteoblasts [23,24], we investigated the effects of Y1 receptor modulation on the activation of ERK signaling as well as p38 signaling. Treatment with Dex or [Leu^31^, Pro^34^]-NPY alone resulted in a reduction of ERK phosphorylation (Figure 5C), and [Leu^31^, Pro^34^]-NPY enhanced the negative effects of Dex on ERK phosphorylation. In contrast, blockade of Y1 receptor by shRNA or BIBP3226 reversed the downregulation of phosphorylated ERK expression induced by Dex. Unlike ERK signaling, the expression of p38 was not affected by glucocorticoid or Y1 receptor modulation (Figure 5D). 

**Figure 6:**
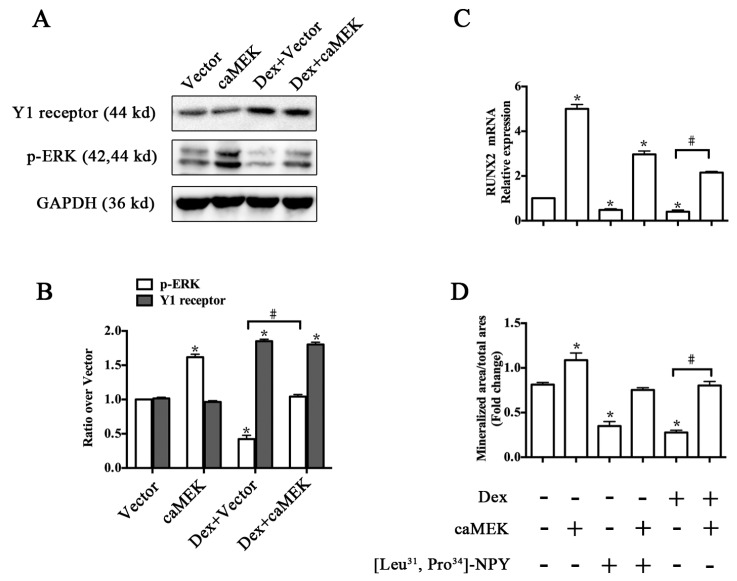
ERK signaling activation by constitutive active mutant of *MEK1* (caMEK) attenuated the inhibitory effects of Dex on osteoblast differentiation in MC3T3-E1 cells; (**A**,**B**) caMEK increased the baseline and Dex-modulated level of phosphorylated ERK expression, but did not affect the levels of Y1 receptor; (**C**) Activation of ERK signaling alleviated the inhibitory effects of Dex or [Leu^31^, Pro^34^]-NPY on runt-related transcription factor 2 (RUNX2) expression and (**D**) on mineralization in MC3T3-E1 cells; MC3T3-E1 cells treated with or without [Leu^31^, Pro^34^]-NPY, Dex, and caMEK transfection were cultured in osteogenic differentiation media for one day or 21 days; Mineralization of MC3T3-E1 cells was determined by Alizarin Red S staining; * *p* < 0.05 (compared to vehicle); # *p* < 0.05 (compared to Dex); EKR: extracellular signal-regulated kinases; GAPDH: glyceraldehyde 3-phosphate dehydrogenase; Veh: vehicle; Dex: dexamethasone.

We then investigated whether ERK signaling was involved in Y1 receptor-mediated suppression of osteoblast differentiation induced by glucocorticoid. caMEK transfection significantly activated the ERK phosphorylation, but did not affect Y1 receptor levels in cell cultures (Figure 6A,B). Activation of ERK signaling pathway attenuated the inhibitory effects of Dex or [Leu^31^, Pro^34^]-NPY on RUNX2 expression and mineralization of MC3T3-E1 cells (Figure 6C,D). caMEK transfection also attenuated the glucocorticoid-induced apoptosis of MC3T3-E1 cells (data not shown). 

## 3. Discussion 

In this study, we found that upregulation of Y1 receptor expression was linked to Dex-induced suppression of osteoblastic differentiation in MC3T3-E1 cells. Excessive use of glucocorticoids damaged the function of bone cells [4,6], and the NPY system was found to play a negative role in the regulation of bone remodeling and osteoblast activity [9,12,17,25]. A recent study has suggested that NPY signaling mediated glucocorticoid-induced osteoporosis in mice [20]. The Y1 receptor, one of the most important receptors for NPY, was expressed in osteoblasts and participated in the regulation of bone metabolism [11,12]. However, the role of Y1 receptor signaling in the process of glucocorticoid-induced osteoporosis has not been investigated. This study revealed for the first time that Y1 receptor expression was upregulated by glucocorticoids in osteoblastic MC3T3-E1 cells. Furthermore, Y1 receptor signaling mediated the deleterious effects of glucocorticoid on osteoblast differentiation through the ERK signaling pathway. The obtained results provide novel insights for understanding the molecular mechanisms by which glucocorticoids caused damage on proliferation and differentiation of osteoblasts. Notably, knockdown of the Y1 receptor is an alternative method to attenuate the deleterious effects of glucocorticoids on osteoblastic cells. 

MC3T3-E1 cells, derived from C57/BL mouse calvaria, were pre-osteoblasts that could differentiate into osteoblasts and produced mineralized matrix in osteogenic media containing ascorbic acid and β-glycerophosphate. Dexamethasone (Dex), one of the synthetic glucocorticoids, is widely used in clinical practice and exerts bi-directional effects on bone formation. A low dose of Dex (≤10^−8^ M) upregulated the expression of osteogenic genes and enhanced bone formation, whereas a high dose of Dex (>10^−8^ M) significantly inhibited osteogenic differentiation and mineralization capacity in osteoblasts [26]. Suppression of osteoblast proliferation and differentiation was considered to be a critical factor for glucocorticoid-induced bone loss [3,26]. However, the precise molecular mechanism by which glucocorticoid impaired the function of osteoblasts remained unknown.

Published studies have shown that the knockout of the Y1 receptor increased bone formation in C57/BL mice through the peripheral nervous system [12], whereas Y2 receptor deficiency resulted in high bone formation through the central nervous system [13]. Mice with germ-line or osteoblast-specific deletion of the Y1 receptor displayed high bone mass phenotype due to a high osteogenic capacity of bone marrow stromal cells and enhanced osteoblast activity [11,12]. In this study, the expression of Y1 receptor was confirmed in osteoblastic MC3T3-E1 cells at both the mRNA and protein levels. MC3T3-E1 cells treated with 10^−7^ M Dex displayed enhanced Y1 receptor expression in association with reduced osteogenic gene expressions. Blockade of Y1 receptor by shRNA or its antagonist BIBP3226 alleviated the deleterious effects of Dex on proliferation and differentiation of osteoblastic cells, while agonist [Leu^31^, Pro^34^]-NPY aggravated the negative effects of Dex. These results suggested that activation of Y1 receptor signaling was responsible for glucocorticoid-induced suppression of osteoblast activity. Receptor activator of nuclear factor kappa-B ligand (RANKL) and osteoprotegerin (OPG) were osteoblast-derived proteins, and the balance of RANKL and OPG was essential for bone homeostasis. RANKL stimulates osteoclastogenesis, while OPG inhibits osteoclastogenesis as the decoy receptor for RANKL. Glucocorticoids increased RANKL/OPG ratio in osteoblasts, which was one of the mechanisms for increased bone resorption and glucocorticoid-induced bone loss [27]. In this study, [Leu^31^, Pro^34^]-NPY increased the baseline and Dex-induced RANKL/OPG ratio in osteoblastic cells, whereas BIBP3226 had the opposite effects. The modulation of RANKL/OPG ratio by the Y1 receptor also suggested the potential involvement of the Y1 receptor in bone resorption. 

To explore the mechanisms by which the Y1 receptor mediated the induction of Dex mentioned above, we focused on the response of ERK and p38 signaling to Y1 receptor modulation. The ERK and p38 signaling pathways mediate various biological reactions in osteoblasts [23,24]. The present study showed that ERK signaling was inactivated in Y1 receptor-mediated Dex suppression of osteoblastic activity, whereas p38 signaling expression was not affected in this process. ERK signaling promoted the mitogenic reaction of osteoblastic cells [28], and glucocorticoid was known to inhibit osteogenic differentiation through inactivation of ERK signaling osteoblasts [29]. Moreover, ERK signaling was reported to mediate the mitogenic reaction of NPY in non-osteoblastic cells [30,31], and the Y1 receptor regulated the activation of ERK signaling in HEK293 cells [32], suggesting a regulatory role of Y1 receptor in ERK signaling. In this study, treatment with Dex or Y1 receptor agonist inactivated ERK signaling, while blockade of Y1 receptor attenuates Dex-induced inactivation of ERK signaling. Activation of ERK signaling by caMEK did not affect the level of Y1 receptor, but attenuated the inhibitory effects of Dex or [Leu^31^, Pro^34^]-NPY on the osteoblastic activity in MC3T3-E1 cells. These results verified the involvement of ERK signaling in osteogenesis, and further suggested that the Y1 receptor, through targeting ERK signaling, played a role in glucocorticoid-induced suppression of osteoblast activity. 

The application of glucocorticoid receptor (GR) blocker RU486 abolished the upregulation of Y1 receptor expression in cell cultures after Dex treatment. It has been demonstrated that the glucocorticoid receptor is the master modulator that mediates the biological effects of glucocorticoid on the osteoblasts [26]. Loss of glucocorticoid receptor function protected osteoblasts against glucocorticoid-induced apoptosis, suppression of proliferation, and differentiation [33]. Our results validated that GC-induced upregulation of Y1 receptor expression was triggered through binding to the glucocorticoid receptor. However, there are few studies investigating the precise molecular mechanisms by which GC promoted the Y1 receptor expression, and further studies are needed. 

## 4. Materials and Methods

### 4.1. Cell Culture and Treatments 

The pre-osteoblast cell line MC3T3-E1 (subclone 4), obtained from ATCC, was cultured in α-MEM (#SH30265; Hyclone, GE Healthcare Life Sciences, Pittsburgh, PA, USA) medium containing 10% fetal bovine serum (#10099; Gibco, Thermo Fischer Scientific, Bartlesville, OK, USA). Cell lines have been authenticated by short-tandem repeat (STR) analysis (ACTG Inc, Wheeling, IL, USA), and the cells were negative for mycoplasma as routinely detected by PlasmoTest (InvivoGen, SanDiego, CA, USA). MC3T3-E1 cells were initially cultured in six-well plates (6 × 10^4^ cells/well) and incubated at 37 °C (5% CO_2_/95% air). Induction of osteogenic differentiation was performed as previously described [34]. After growing to 70% confluence, MC3T3-E1 cells were cultured in an osteogenic differentiation medium supplemented with 4 mM β-glycerophosphate (#G9891; Sigma-Aldrich, St. Louis, MO, USA) and 25 μg/mL ascorbic acid (#A4403; Sigma-Aldrich). Dexamethasone (#D4902; Sigma-Aldrich, final concentration of ethanol, 0.01%, vol/vol) at different concentrations was then added to the osteogenic differentiation medium for one day or 21 days. The culture medium was replaced every three days. Under certain circumstances, MC3T3-E1 cells were treated with or without 10^−5^ M RU486 (#M8046; Sigma-Aldrich), 10^−7^ M [Leu^31^, Pro^34^]-NPY (#1176; Tocris Bioscience, Bristol, UK), and 10^−7^ M BIBP3226 (#203842; Santa Cruz Biotechnology Inc., Santa Cruz, CA, USA) in a culture medium containing 10^−7^ M Dex.

### 4.2. Transfection of Y1 Receptor shRNA Plasmid 

The shRNA plasmid targeting Y1 receptor gene was purchased from Santa Cruz (#36098-SH). According to the manufacturer’s instructions, subconfluent MC3T3-E1 cells were transfected with Y1 receptor shRNA plasmid or scrambled control by using Lipofectamine 2000 (#11668; Invitrogen, Thermo Fischer Scientific, Bartlesville, OK, USA). Knockdown of Y1 receptor gene expression was confirmed by real-time PCR and Western blot analysis. For selection of stably transfected cells, Puromycin (4 μg/mL, #A11138; Gibco) was added to the culture for five days. 

### 4.3. Transfection of Active Mutant of MEK-1 cDNAs

To activate the ERK signaling pathway, the cDNAs coding constitutively active MEK-1 mutant (S218D/S222D; caMEK) were subcloned into pUSE vectors [35,36]. MC3T3-E1 cells were transfected with caMEK or empty vectors by the lipofection method. Stable populations were selected with Geneticin (500 μg/mL, #10131; Life Technologies, Carlsbad, CA, USA).

### 4.4. Cell Proliferation and Viability Assay 

The cell proliferation and viability rates were determined by using a cell counting kit (CCK-8, Dojindo Laboratories, Rockville, MD, USA), according to the manufacturer’s instructions. Briefly, MC3T3-E1 cells seeded in 96-well plates (5 × 10^3^ cells/well) were incubated in osteogenic differentiation media in the presence or absence of 10^−7^ M dexamethasone for 24 h. A 10-μL volume of CCK-8 reagent was then added to each well. After incubating the mixture for another 1 h at 37 °C, the absorbance value of each sample was spectrophotometrically determined at a wavelength of 450 nm using a microplate reader (Bio-Rad, Hercules, CA, USA). Cell proliferation and viability rate were expressed as fold changes relative to the vehicle groups. All experiments were conducted in triplicate. 

### 4.5. Real-Time PCR Analysis 

Total RNA was isolated using Trizol reagent (#15596; Invitrogen) and quantified by absorbance measurement at the wavelength of 260 nm. To obtain cDNA, quantified RNA was reverse transcribed by using TaKaRa PCR Kit (TaKaRa Biotechnology, Beijing, China). The specific primers for mouse Y1 receptor, NPY, RUNX2, OCN, OPG and RANKL, as well as their gene numbers, are shown in Table 1. An ABI 7500 system (Applied Biosystems, Foster City, CA, USA) was then used to perform quantitative real-time PCR using SYBR Premix Ex Taq (TaKaRa Biotechnology) as previously described [34]. The specificity of the transcript amplification was verified by a melting curve analysis. The fold change in expression of the target gene was quantified using the 2^−ΔΔ*C*t^ methods after normalization to β-actin. 

### 4.6. Western Blot Analysis 

Total cell lysate was collected using ice-cold RIPA lysis buffer (Beyotime, Haimen, China) according to the manufacturer’s instruction. The protein content was then determined using Bicinchoninic Acid (BCA) Protein Determination Kit (Beyotime) with BSA as a standard protein. Each sample of 40 µg total protein was loaded onto 8%–12% SDS-PAGE and electrotransferred to a polyvinylidene difluoride (PVDF) membrane. Membranes were blocked with 5% nonfat dry milk (diluted in TBST(50 mM Tris-HCl, pH 7.5, 150 mM NaCl, 0.05% (*v*/*v*) Tween 20)) for 2 h at room temperature before incubation with primary antibodies for Y1 receptor (#35336; Abcam), phosphorylated ERK (#9101; CST, San Antonio, TX, USA), phosphorylated p38 (#9211; CST), total ERK (#4695; CST), total p38 (#8690; CST), Cleaved Caspase-3 (#9664; CST), Cleaved Caspase-9 (#9509; CST), and GAPDH (#AG019; Beyotime) at 4 °C overnight. The membranes were then washed thrice with TBST and incubated with HRP-conjugated secondary antibody (Beyotime, China) for 1 h. The target protein expression was detected using the Western Chemiluminescent HRP Substrate Kit (Millipore, Billerica, MA, USA), with GAPDH as the endogenous control. Quantitative densitometric values of the detected bands were quantified using the NIH Image J Software.

### 4.7. Alizarin Red S Staining

Mineralization assay for MC3T3-E1 cells was performed by using Alizarin Red S staining (#A5533; Sigma-Aldrich), as previously described [34]. Briefly, cells were incubated in the osteogenic differentiation media containing 4 mM β-glycerophosphate and 25 μg/mL ascorbic acid for 21 days. The number of mineralized nodules and the areas of mineralized matrix in each well were analyzed as previously described [34]. Six fields were randomly selected as the region of interest for each well, and then the samples were observed under a dissecting microscope at 30× magnification. The mineralized nodes showing positive Alizarin Red staining were identified and counted under a microscope. The areas of mineralized matrix were then quantified using Image-Pro Plus software (Media Cybernetics, Silver Spring, MD, USA). The relative area of mineralized matrix was determined as mineralized area/total area × 100%. Fold changes were expressed as treatment group/vehicle group. 

### 4.8. Statistical Analysis 

We performed all the independent experiments at least three times and presented the data as the mean ± SEM. Statistical differences were determined by the appropriate Student’s *t*-test or one-way ANOVA followed by a post hoc Fisher’s least significant difference (LSD) test. Statistical analyses were performed using SPSS software (SPSS 18.0; SPSS, Chicago, IL, USA), with *p* values less than 0.05 considered statistically significant. 

## 5. Conclusions

In this study, we demonstrated that Y1 receptor signaling was regulated by glucocorticoid treatment. Upregulation of the Y1 receptor by glucocorticoid led to inactivation of ERK signaling, thus decreasing osteoblastic proliferation and differentiation in MC3T3-E1 cells. Blockade of the Y1 receptor can effectively antagonize the inhibitory effects of Dex on osteoblastic cells. The present study is an effort to understand the complex molecular mechanisms of glucocorticoid-induced osteoporosis, and to provide new insights into Y1 receptor regulation of the glucocorticoid-induced suppression of proliferation and differentiation in osteoblasts. This study further suggests the great value of Y1 receptor downregulation in promoting osteoblast-mediated bone formation and even reversing glucocorticoid-induced osteoporosis.

## Figures and Tables

**Table 1:** Primers used for real-time PCR.

Name	Primer Sequence (5′-3′) Sense/Antisense	GenBank Number
Y1 Receptor [10]	CTCGCTGGTTCTCATCGCTGTGGAACGG	NM_010934
	GCGAATGTATATCTTGAAGTAG	
NPY	CTCGTGTGTTTGGGCATTC	NM_023456
	TAGTGTCGCAGAGCGGAGTA	
RUNX2 [34]	GACGAGGCAAGAGTTTCACC	NM_009820
	GGACCGTCCACTGTCACTTT	
OCN	CAAGCAGGGAGGCAATAAGG	NM_007541
	CGTCACAAGCAGGGTTAAGC	
OPG [34]	AGCTGCTGAAGCTGTGGAA	NM_008764
	GGTTCGAGTGGCCGAGAT	
RANKL [34]	GAAGGCTCATGGTTGGATGT	NM_011613
	GTAGCCCAAGGGTATTTCAG	
β-actin [34]	TGACAGGATGCAGAAGGAGA	NM_007393
	CGCTCAGGAGGAGCAATG	

NPY: neuropeptide Y; RUNX2: runt-related transcription factor 2; OCN: osteocalcin; RANKL: receptor activator of nuclear factor kappa-B ligand.

## References

[1] Compston J. (2010). Management of glucocorticoid-induced osteoporosis. Nat. Rev. Rheumatol..

[2] Tait A.S., Butts C.L., Sternberg E.M. (2008). The role of glucocorticoids and progestins in inflammatory, autoimmune, and infectious disease. J. Leukoc. Biol..

[3] Migliaccio S., Brama M., Fornari R., Greco E.A., Spera G., Malavolta N. (2007). Glucocorticoid-induced osteoporosis: an osteoblastic disease. Aging Clin. Exp. Res..

[4] Weinstein R.S., Jilka R.L., Parfitt A.M., Manolagas S.C. (1998). Inhibition of osteoblastogenesis and promotion of apoptosis of osteoblasts and osteocytes by glucocorticoids: Potential mechanisms of their deleterious effects on bone. J. Clin. Investig..

[5] Zaidi M., Sun L., Robinson L.J., Tourkova I.L., Liu L., Wang Y., Zhu L.L., Liu X., Li J., Peng Y. (2010). ACTH protects against glucocorticoid-induced osteonecrosis of bone. Proc. Natl. Acad. Sci. USA.

[6] Henneicke H., Gasparini S.J., Brennan-Speranza T.C., Zhou H., Seibel M.J. (2014). Glucocorticoids and bone: local effects and systemic implications. Trends Endocrinol. Metab..

[7] Xia X., Kar R., Gluhak-Heinrich J., Yao W., Lane N.E., Bonewald L.F., Biswas S.K., Lo W.K., Jiang J.X. (2010). Glucocorticoid-induced autophagy in osteocytes. J. Bone Miner. Res..

[8] Li J., Zhang N., Huang X., Xu J., Fernandes J.C., Dai K., Zhang X. (2013). Dexamethasone shifts bone marrow stromal cells from osteoblasts to adipocytes by C/EBPalpha promoter methylation. Cell Death Dis..

[9] Shi Y.C., Baldock P.A. (2012). Central and peripheral mechanisms of the NPY system in the regulation of bone and adipose tissue. Bone.

[10] Lundberg P., Allison S.J., Lee N.J., Baldock P.A., Brouard N., Rost S., Enriquez R.F., Sainsbury A., Lamghari M., Simmons P. (2007). Greater bone formation of Y2 knockout mice is associated with increased osteoprogenitor numbers and altered Y1 receptor expression. J. Biol. Chem..

[11] Lee N.J., Doyle K.L., Sainsbury A., Enriquez R.F., Hort Y.J., Riepler S.J., Baldock P.A., Herzog H. (2010). Critical role for Y1 receptors in mesenchymal progenitor cell differentiation and osteoblast activity. J. Bone Miner. Res..

[12] Baldock P.A., Allison S.J., Lundberg P., Lee N.J., Slack K., Lin E.J., Enriquez R.F., McDonald M.M., Zhang L., During M.J. (2007). Novel role of Y1 receptors in the coordinated regulation of bone and energy homeostasis. J. Biol. Chem..

[13] Baldock P.A., Sainsbury A., Couzens M., Enriquez R.F., Thomas G.P., Gardiner E.M., Herzog H. (2002). Hypothalamic Y2 receptors regulate bone formation. J. Clin. Investig..

[14] Baldock P.A., Lee N.J., Driessler F., Lin S., Allison S., Stehrer B., Lin E.J., Zhang L., Enriquez R.F., Wong I.P. (2009). Neuropeptide Y knockout mice reveal a central role of NPY in the coordination of bone mass to body weight. PLoS ONE.

[15] Sousa D.M., Baldock P.A., Enriquez R.F., Zhang L., Sainsbury A., Lamghari M., Herzog H. (2012). Neuropeptide Y Y1 receptor antagonism increases bone mass in mice. Bone.

[16] Igwe J.C., Jiang X., Paic F., Ma L., Adams D.J., Baldock P.A., Pilbeam C.C., Kalajzic I. (2009). Neuropeptide Y is expressed by osteocytes and can inhibit osteoblastic activity. J. Cell. Biochem..

[17] Lee N.J., Nguyen A.D., Enriquez R.F., Doyle K.L., Sainsbury A., Baldock P.A., Herzog H. (2011). Osteoblast specific Y1 receptor deletion enhances bone mass. Bone.

[18] Laborie C., Bernet F., Kerckaert J.P., Maubert E., Lesage J., Dupouy J.P. (1995). Regulation of neuropeptide Y and its mRNA by glucocorticoids in the rat adrenal gland. Neuroendocrinology.

[19] Myrsen-Axcrona U., Karlsson S., Sundler F., Ahren B. (1997). Dexamethasone induces neuropeptide Y (NPY) expression and impairs insulin release in the insulin-producing cell line RINm5F: Release of NPY and insulin through different pathways. J. Biol. Chem..

[20] Wang F.S., Lian W.S., Weng W.T., Sun Y.C., Ke H.J., Chen Y.S., Ko J.Y. (2016). Neuropeptide Y mediates glucocorticoid-induced osteoporosis and marrow adiposity in mice. Osteoporos. Int..

[21] Bai J., Meng Z. (2010). Expression of caspase and apoptotic signal pathway induced by sulfur dioxide. Environ. Mol. Mutagen..

[22] Sheen J.M., Chen Y.C., Hsu M.H., Tain Y.L., Huang Y.H., Tiao M.M., Li S.W., Huang L.T. (2016). Melatonin Alleviates Liver Apoptosis in Bile Duct Ligation Young Rats. Int. J. Mol. Sci..

[23] Chen C., Koh A.J., Datta N.S., Zhang J., Keller E.T., Xiao G., Franceschi R.T., D’Silva N.J., McCauley L.K. (2004). Impact of the mitogen-activated protein kinase pathway on parathyroid hormone-related protein actions in osteoblasts. J. Biol. Chem..

[24] Bianchi E.N., Ferrari S.L. (2009). Beta-arrestin2 regulates parathyroid hormone effects on a p38 MAPK and NFkappaB gene expression network in osteoblasts. Bone.

[25] Horsnell H., Baldock P.A. (2016). Osteoblastic Actions of the Neuropeptide Y System to Regulate Bone and Energy Homeostasis. Curr. Osteoporos. Rep..

[26] Rauch A., Seitz S., Baschant U., Schilling A.F., Illing A., Stride B., Kirilov M., Mandic V., Takacz A., Schmidt-Ullrich R. (2010). Glucocorticoids suppress bone formation by attenuating osteoblast differentiation via the monomeric glucocorticoid receptor. Cell Metab..

[27] Hofbauer L.C., Zeitz U., Schoppet M., Skalicky M., Schuler C., Stolina M., Kostenuik P.J., Erben R.G. (2009). Prevention of glucocorticoid-induced bone loss in mice by inhibition of RANKL. Arthritis Rheum..

[28] Miguel S.M., Namdar-Attar M., Noh T., Frenkel B., Bab I. (2005). ERK1/2-activated de novo Mapkapk2 synthesis is essential for osteogenic growth peptide mitogenic signaling in osteoblastic cells. J. Biol. Chem..

[29] Wu R.W., Lin T.P., Ko J.Y., Yeh D.W., Chen M.W., Ke H.C., Wu S.L., Wang F.S. (2011). Cannabinoid receptor 1 regulates ERK and GSK-3beta-dependent glucocorticoid inhibition of osteoblast differentiation in murine MC3T3-E1 cells. Bone.

[30] Pellieux C., Sauthier T., Domenighetti A., Marsh D.J., Palmiter R.D., Brunner H.R., Pedrazzini T. (2000). Neuropeptide Y (NPY) potentiates phenylephrine-induced mitogen-activated protein kinase activation in primary cardiomyocytes via NPY Y5 receptors. Proc. Natl. Acad. Sci. USA.

[31] Cho Y.R., Kim C.W. (2004). Neuropeptide Y promotes beta-cell replication via extracellular signal-regulated kinase activation. Biochem. Biophys. Res. Commun..

[32] Lecat S., Belemnaba L., Galzi J.L., Bucher B. (2015). Neuropeptide Y receptor mediates activation of ERK1/2 via transactivation of the IGF receptor. Cell Signal..

[33] Heitzer M.D., Wolf I.M., Sanchez E.R., Witchel S.F., DeFranco D.B. (2007). Glucocorticoid receptor physiology. Rev. Endocr. Metab. Disord..

[34] Zhu C., Zheng X.F., Yang Y.H., Li B., Wang Y.R., Jiang S.D., Jiang L.S. (2016). LGR4 acts as a key receptor for R-spondin 2 to promote osteogenesis through Wnt signaling pathway. Cell Signal..

[35] Lemieux E., Boucher M.J., Mongrain S., Boudreau F., Asselin C., Rivard N. (2011). Constitutive activation of the MEK/ERK pathway inhibits intestinal epithelial cell differentiation. Am. J. Physiol. Gastrointest. Liver Physiol..

[36] Gopalbhai K., Jansen G., Beauregard G., Whiteway M., Dumas F., Wu C., Meloche S. (2003). Negative regulation of MAPKK by phosphorylation of a conserved serine residue equivalent to Ser212 of MEK1. J. Biol. Chem..

